# Flowering induction in cassava using photoperiod extension premature pruning and plant growth regulators

**DOI:** 10.1371/journal.pone.0292385

**Published:** 2023-10-05

**Authors:** Alexandra Damasceno Santos, Massaine Bandeira e Sousa, Alfredo Augusto Cunha Alves, Eder Jorge de Oliveira

**Affiliations:** 1 Universidade Federal do Recôncavo da Bahia, Cruz das Almas, Bahia, Brasil; 2 Embrapa Mandioca e Fruticultura, Cruz das Almas, Bahia, Brasil; University of Agricultural Sciences, INDIA

## Abstract

Cassava (*Manihot esculenta* Crantz) is a vital crop for food and economic security in many regions of the world. Despite the economic and social importance of cassava, challenges persist in developing superior varieties that meet the needs of farmers in terms of agronomic performance, nutritional quality, and resistance to pests and diseases. One of the main obstacles for genetic improvement is the lack of synchronization in flowering and the abortion of young flowers, making planned crosses and progeny production difficult. Therefore, the aim of this study was to evaluate the effect of photoperiod, premature pruning, and growth regulators on cassava flowering under low-altitude conditions in Brazil. Eight cassava clones with contrasting flowering capacity were assessed in Cruz das Almas, Bahia, using two photoperiods (ambient condition and extended photoperiod with red light for 12 hours), premature pruning at the first and second branching levels (with and without pruning), and the application of growth regulators: 0.5 mM 6-benzyladenine (BA) and 4.0 mM silver thiosulfate (STS) (with and without). Plots were assessed weekly for the number of female (NFF) and male (NMF) flowers, height of the first branching (H1B, in cm), number of days to the first branching (ND1B), and the number of branching events up to 240 days after planting (NOB). The extended photoperiod did not promote an increase in the number of flowers but allowed for precocity in cassava flowering, reducing the onset of flowering by up to 35 days, and significantly increasing the number of branches, which is closely related to flowering. The use of pruning and plant growth regulators (PGR) resulted in an increase in NFF from 2.2 (control) to 4.6 and NMF from 8.1 to 21.1 flowers. Therefore, under hot and humid tropical conditions at low altitudes in the Recôncavo of Bahia, manipulating the photoperiod and using premature pruning and plant growth regulators can accelerate cassava flowering, benefiting genetic improvement programs.

## Introduction

Cassava (*Manihot esculenta* Crantz) is an important source of carbohydrates in the diet of about 800 million people [1] especially in developing countries. More than half of world cassava production in 2019 came from Nigeria, Democratic Republic of Congo, Thailand and Ghana, which produced 59.19, 40.05, 31.07, and 22.44 million tons of roots, respectively [2].

Brazil has the 5^th^ position with annual production of 17.49 million tons in 1.19 million hectares harvested [2]. In 2012, the global trade of cassava products and the growth of the crop on the African continent enabled the crop to reach record levels of root production [1]. However, in Brazil there are still huge yield gaps, since the national average productivity is 14.70 t.ha^-1^, far below the yield potential obtained from new cultivars (27.50 t.ha^-1^ in annual cycle) [3].

Low cassava yield can be attributed to various factors, such as the lack of appropriate fertilization, which can make the crop more susceptible to pests and diseases due to inadequate plant nutrition for proper growth and development [4]. Additionally, insufficient technological input associated with planting methods, including the use of low-quality land, limited crop rotation, obsolete and low-yielding cultivars, and unpredictable rainfall patterns, also contribute to low root yields [5]. One of the most commonly employed approaches to address these issues, regardless of farmers’ level of technological expertise, is to use improved varieties with high yield potential and resistance to adverse factors. Breeding programs are continuously developing new cultivars with improved agronomic performance, nutritional quality, and resistance to pests and diseases [6, 7].

In the commercial cultivation system, cassava is clonally propagated, resulting in no genetic variation across generations. To overcome this limitation, conventional breeding techniques can be used to obtain seeds through self-fertilization or controlled crosses, which are then used to select superior genotypes for further breeding through classical and advanced approaches [8].

Cassava is a monoecious plant, with female flowers formed in the lower part of the inflorescence and smaller, more numerous male flowers on the upper part [9, 10]. However, planning crosses for cassava breeding is challenging due to the wide variability in traits related to flowering onset, intensity, and duration [11, 12]. Additionally, farmers’ preference for erect plant architecture and absence of lateral branching makes it difficult to use these genotypes as parents, as branching is highly correlated with flowering [13, 14]. Therefore, the use of these clones as parents for population improvement is greatly compromised by the absence of forking and consequently flowering.

In plants, the transition from vegetative to reproductive growth is triggered by genetic, endogenous (plant hormones and nutritional status), and environmental factors, such as solar radiation, photoperiod (day length), water availability, and temperature [10–12, 15–17]. At the molecular level, genes such as FT (flowering locus T), SOC1 (suppressor of CO1 gene overexpression), and LFY (leafy) are involved in flowering signaling and photoperiod sensitivity in cassava [18–20]. The FT protein results from a cellular signal initiated by the CO protein from leaves to the apical meristem and florigen [21]. Florigen is a systemic signal generated by sunlight captured by the leaves, regulated primarily by changes in photoperiod during the day. This signal is transported through the phloem to the apical meristem, inducing gene expression responsible for flowering [22]. The extension of photoperiod using red light has been shown to reduce the time to initiation of flowering, confirming the sensitivity of the *MeFT2* gene (a member of the phosphatidylethanolamine-binding protein family, which includes FT) to photoperiod [17].

Auxins, gibberellins, abscisic acid, cytokinins, and ethylene are plant hormones that can directly or indirectly affect metabolic pathways at different stages of plant growth [23]. Abscisic acid, jasmonates, brassinosteroids, polyamines, and salicylic acid are also involved in various physiological processes and can have analogous or antagonistic actions to the primary plant hormones. These hormones can have a synergistic or antagonistic interaction in the regulation of the flowering pathway, which means they can either work together or hinder the regulation of factors that induce flowering [24], that is, they can act in a cooperative way, or hinder the regulation of factors that induce flowering. Therefore, the application of exogenous plant growth regulators (PGRs) has become an alternative for inducing flowering in cassava crops [11, 16, 25].

The most commonly used PGR in cassava is 6-benzyladenine [26, 27]. [28] reported that cytokinin can promote sex determination during floral development by modifying the apical meristem of male flowers, inducing the formation of the gynecium and the pistil in *Plukenetia volubilis* (Euphorbiaceae). This sexual modification of flowers suggests that genes responsible for sexual differentiation undergo suppression or activation through a signal transduction mechanism that modifies endogenous hormone levels, as observed in *Jatropha curcas* [29, 30]. Studies have shown that 6-benzyladenine can also inhibit lipid peroxidation, preserve membrane integrity in plant tissues [31] increase antioxidant capacity, and delay leaf senescence in *Brassica campestris* species [32].

Another PGR commonly used in cassava is silver thiosulfate (STS), which is used as an ethylene inhibitor during the process of abscission and floral senescence [33]. Silver ions (Ag+) have been successfully used to inhibit the production or action of ethylene by acting as a competitive inhibitor of the binding between ethylene and its receptor. Ag+ can also inhibit ethylene synthesis by blocking the autocatalysis present in flowers and fruits [34]. The STS ionic complex is formed by the association between a solution of silver nitrate and sodium thiosulfate, which has greater mobility in the plant and less phytotoxicity than silver nitrate [35].

Premature pruning of lateral shoots has also shown promise as a flowering induction technique in cassava [11]. This technique favors energy transport to the apical meristem, inducing flowering while also increasing the light and air uptake by the plant, providing support for the maintenance of the photosynthetic process [36]. Additionally, the use of PGR associated with premature pruning at different branching levels has promoted a significant interaction between the technique and hormone signaling genes, inducing flowering in cassava [25].

The development of effective methodologies for floral induction is crucial for intercontinental breeding programs, especially considering the diverse environmental conditions outside Brazil. In West Africa, researchers have found that clones with different flowering times, adapted to the region and high altitudes, had higher flower and fruit production when subjected to STS, BA, and pruning [25]. Similarly, positive results have been reported in Colombia and the United States using photoperiod extension and regulators to induce flowering [11, 37]. However, the varying temperature, photoperiod, and altitude in these regions pose challenges for developing effective methodologies that suit Brazil’s climatic conditions [11, 16, 26].

In a study conducted in Brazil [38], found that the flower abortion rate could be as high as 88%, emphasizing the importance of developing effective methodologies for floral induction in the country. Moreover, the diverse flowering patterns found in cassava germplasm make it difficult to generate new segregant populations and recombine elite clones [14, 17, 39, 40].

To address these challenges, it is necessary to understand the effect of photoperiod extension, premature pruning of lateral shoots, and growth regulators in cassava’s flowering induction, specifically under the climatic conditions of the Coastal Tablelands of Northeast Brazil. Such knowledge would enable the development of effective and adapted techniques for inducing cassava flowering, which would lead to efficient planning of controlled crosses and increase the genetic gains in breeding programs by increasing the number of recombinant individuals in progenies. Therefore, this study aims to evaluate the effect of extending the photoperiod using red light, premature pruning of lateral branches, and the application of BA and STS in the flowering induction in cassava.

## Material and methods

### Germplasm and experimental evaluation

We evaluated eight different cassava genotypes belonging to the Cassava Genebank (BAG) of Embrapa Mandioca e Fruticultura, each with different flowering intensities and patterns as described in Table 1. The field experiment was conducted in the experimental area of Embrapa Mandioca and Fruticultura in Cruz das Almas, Bahia, Brazil, from October 2020 to December 2021. The region has an equatorial Af climate, with an average annual rainfall of 1170 mm, ranging from 900 to 1300 mm, an average annual temperature of 23.9°C, according to Köppen and Geiger classification, and an average annual relative humidity of 81% [41].

**Table 1:** Flowering characteristics of cassava genotypes evaluated under different photoperiod treatment, premature pruning and plant growth regulators.

Genotypes	Genotype	Flowering	Intensity
BRS CS01	Cultivar	Early	Abundant
BRS Tapioqueira	Cultivar	Late	Abundant
BRS Kiriris	Cultivar	Late	Medium
BRS Novo Horizonte	Cultivar	Late	Medium
BR-14-006-02	Improved clone	Absent	Not known
BR-14-010-11	Improved clone	Early	Low
BR-17-006-62	Improved clone	Late	Low
BR-17-012-59	Improved clone	Late	Low

We obtained data for rainfall and minimum, maximum, and average monthly temperature during the experiment from the meteorological station of Embrapa Mandioca and Fruticultura (S1 Fig). The local photoperiod was approximately 12 hours, with small variations throughout the year and an amplitude of about 40 minutes between December and May.

The experiment was conducted using a randomized complete block design with three replicates per treatment in a split-plot design. The primary plot was constituted by the photoperiod condition (dark night—DN and photoperiod extension—PE). DN represented the control or normal environmental conditions without lights (S2 Fig). For the PE condition, we used 54 50W red light LED reflectors that were turned on at sunset (5 pm) and turned off after sunrise (6 am). The reflectors were fixed in the field at a height of 3 meters and arranged in six rows with a spacing of 9 meters. We installed the two photoperiod conditions with a distance of 16 meters between them to prevent interference of red light on the control treatments. Pruning was carried out on the first and second branches of the cassava genotypes (S3 Fig).

Within each photoperiod condition, we evaluated five treatments: 1) no pruning; 2) pruning in the 1^st^ branching tier (1stBT); 3) pruning in the 2^nd^ branching tier (2nBT); 4) ‘Pruning + PGR’ (plant growth regulator) in the 1stBT; and 5) ‘Pruning + PGR’ in the 2ndBT. For each combination of genotype, photoperiod, and treatment, we evaluated plots consisting of four useful plants in the plot (eight plants in total).

For planting, we used 20 cm long cuttings with an average of eight buds, taken from the middle third of 12-month-old plants with good growth conditions. We followed the standard planting spacing of 0.90 m between rows and 0.80 m between plants in the plot. The cultivation process was carried out according to the recommendations of [42].

### Pruning and application of plant growth regulators

To identify signs of flowering, plants with a minimum height of 60 cm were inspected weekly, and lateral branches measuring 1 to 2 cm long, identified in the apical meristem, were pruned carefully using surgical blades to preserve the integrity of the inflorescence, following the method described by [11]. Lateral branches that developed below the pruning apex, about 10 cm below, were removed periodically.

For the combined pruning treatment, we used two growth regulators: 6-Benzyladenine (BA) 0.5 mM and silver thiosulfate (STS) 4.0 mM. To prepare BA, we mixed 5.7 mL of the commercial product Maxcel (Valent BioSciences, Libertyville, Illinois, USA) with 1000 mL of distilled water [11]. We applied the solution to the inflorescences once a week, in the morning, using a sprayer until the senescence period of the flowers, which typically lasted for two weeks on average. We sprayed approximately 5 ml of the solution per inflorescence. To prepare STS, we slowly added 40 mL of 0.1M silver nitrate solution to 160 mL of 0.1M sodium thiosulfate solution, obtaining 200 mL of 20 mM STS [25]. We then added 200 mL of the 20 mM STS solution to 800 mL of water to obtain 1000 mL of 4 mM STS. We applied STS via petiole to 40 cm below the apex of the plant every 14 days until fruit harvest was completed. To apply the solution, we removed the leaf with a surgical blade and inserted a 15 mL conical Falcon tube (Falcon Brand, Corning, NY, USA) containing 2.5 ml of STS solution. The petioles were left immersed in the solution for 72 hours.

### Phenotyping

Phenotypic data were collected weekly for each individual plant in each plot, including the number of female flowers (NFF) and male flowers (NMF) at the first and second branching level, and traits related to flowering time, such as first branching height (H1B, in cm), number of days to first branching (ND1B), and the number of branching levels up to 240 days after planting (NOB) (analyzed only in the control plots).

### Statistical analysis

An analysis of variance (ANOVA) was performed for all traits using the lme4 package [43]. The initial analysis was performed on the primary plot, taking into account the effect of photoperiod and genotypes as fixed effects. Then, ANOVA was performed for the flower production traits, considering genotype and the interaction between genotypes and treatments (combination of pruning position and the presence or absence of PGR) as random effects, and treatment effect as fixed. Therefore, the phenotypic observation *Y*ik of genotype *i* within treatment *k* was modeled by the equation: $Y_{ik}=\mu +{t_{k}+g}_{i}+{\left(g*t\right)}_{ik}+\epsilon_{ik}$, where μ is the overall mean, *t*k is the fixed treatment effect, *g*_i_ is the random effect of genotype, (*g* * *t*) is the random effect of the interaction between genotypes and treatments, and ∈_ik_ is the residual random effect of genotype *i* within treatment *k*.

To compare the means of the treatments for each genotype, the Tukey test (p ≤ 0.05) was used, and the rstatix package [44] was employed. To compare the means of the treatments overall, the Scott-Knott test of means (p ≤ 0.05) was performed using the ScottKnott package [45]. All the packages were implemented in R software version 4.1.2 [46].

## Results

### Effects of photoperiod on cassava genotype development

The impact of photoperiod treatment on cassava genotype development was analyzed. The results showed significant effects of photoperiod treatment on the number of days to first branching (ND1B), height of first branching (H1B), and number of branching levels (NOB) up to 240 days after planting, but not on the number of female flowers (NFF) and male flowers (NMF) (Table 2).

**Table 2:** Summary of variance analysis for effects of photoperiod length on traits: Number of days to first branching (ND1B), height of first branching (H1B), number of branching tiers (NOB), number of female flowers (NFF), and male flowers (NFM), evaluated in eight cassava genotypes.

Source of variation	DF^a^	Mean Square				
		ND1B	HIB	NOB	NFF	NMF
Genotype (G)	7	136042***	23103.10***	6.39***	18.87^ns^	656.54**
Photoperiod (P)	1	26556**	5315.20*	3.48**	14.88^ns^	647.76^ns^
P × G	7	13936***	3328.10**	0.94**	12.49^ns^	167.08^ns^
Error	131	2436^ns^	942.90^ns^	0.35^ns^	9.38^ns^	223.22^ns^
Average		214.90	146.03	0.87	6.77	30.22
CV^b^		0.48	0.33	1.03	1.71	1.63

^a^Degree of freedom

b: Coefficient of variation

*: Significance: *** = 0.001

** = 0.01; * = 0.05

ns: Not significant

**Fig 1:**
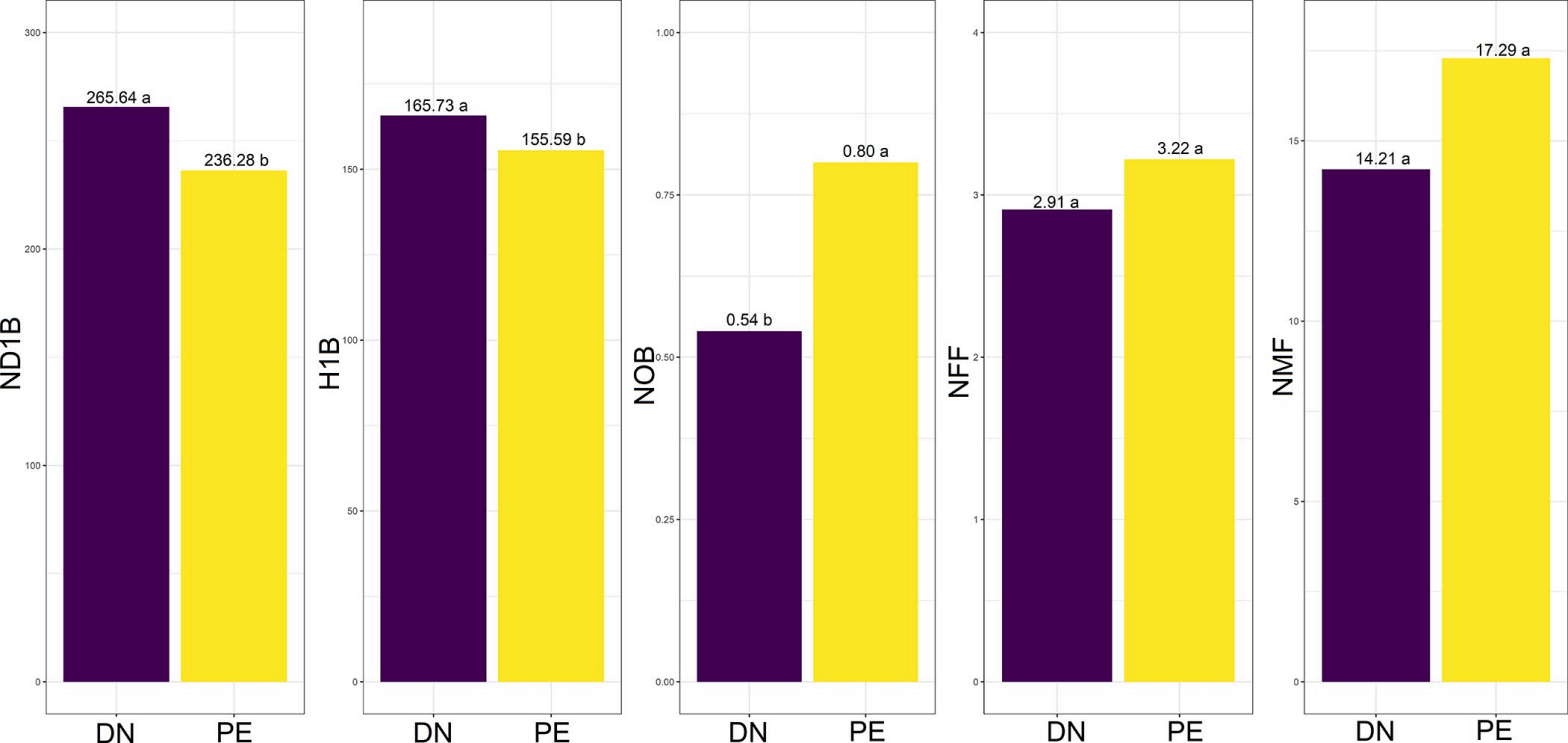
Analysis of means using the Scott-Knott test (p ≤ 0.05) to assess the following variables in eight cassava genotypes, with and without photoperiod extension (PE): Number of days to first branching (ND1B), height of first branching (H1B, in cm), total number of branches (NOB) up to 240 days after planting, as well as the number of female flowers (NFF) and male flowers (NMF).

Fig 1 shows the overall mean among genotypes for the control (DN) and photoperiod extension (PE) treatments. The results indicated a reduction of approximately 12.8 cm in H1B and 35 days for ND1B in the PE treatment, indicating the beneficial effect of photoperiod extension on early branch development in cassava. Moreover, plants subjected to photoperiod extension showed higher NER throughout the evaluations. However, ANOVA did not show a significant increase in the number of female flowers (NFF) and male flowers (NMF) in the PE treatment.

**Fig 2:**
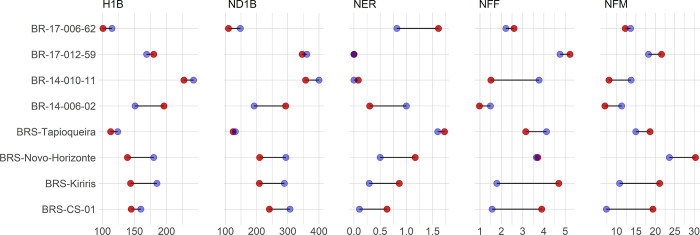
Genotype performance assessed through mean comparison using the Tukey test (p ≤ 0.05) for the following characteristics: Height of the first branch (H1B, in cm), days to first branching (ND1B), number of rows of branches (NOB—assessed up to 240 days after planting), number of female flowers (NFF), and number of male flowers (NMF) under two photoperiod treatments: Dark night (DN), represented in blue, and photoperiod extension (PE), represented in red; * indicates significant differences.

Individual performance analysis revealed that the genotypes BRS Novo Horizonte and BRS Kiriris, characterized by late flowering, demonstrated a significant reduction in the number of days for the beginning of flowering (90 and 81 days, respectively). Conversely, the genotype BRS CS01 maintained its early blooming behavior, with a significant reduction in the number of days for the beginning of flowering by 66 days (Fig 2). The genotype BR-14-006-02, characterized by absent flowering, showed an increase in the number of days for the beginning of ramification and H1B, and a reduction in NOB when exposed to the photoperiod extension.

The genotype BR-17-006-62, characterized by low and late flowering, showed a significant increase in NOB when exposed to the photoperiod extension. Moreover, the genotype BRS Kiriris, which is characterized by medium intensity of flowering, demonstrated the highest increase in NFF in relation to the other genotypes in the treatments with photoperiod extension. On the other hand, the genotypes BR-17-012-59 and BRS Tapioqueira showed no significant effects for the evaluated characteristics when submitted to the photoperiod extension.

### Effect of early pruning and plant growth regulators

To investigate the effects of early pruning and plant growth regulators, the number of male and female flowers at the first two branching tiers (1stBT and 2nBT) of the control plots were analyzed. Initially, the effects of pruning were examined by comparing the number of flowers at the 1stBT of the control plots to those of the pruned plots, which showed significant effects on the number of male and female flowers (p<0.01) (Table 3). However, at the 2ndBT, only the number of female flowers showed a significant effect.

**Table 3:** Summary of variance analysis for effects of premature pruning on traits: Number of female flowers (NFF) and male flowers (NFM), evaluated in eight cassava genotypes.

Source of variation	DF	Mean Square			
		Control 1stBT^a^		Control 2nBT^b^	
		NFF	NMF	NFF	NMF
Block	5	3.16	214.33	4.16	241.50
Genotypes	7	132.4*	652.62***	41.05***	1194.18***
Pruning	4	76.05***	1995.02***	36.12**	380.90^ns^
C^c^ 1: Control vs. Pruning	1	172.40***	7105***	7.40^ns^	687.90^ns^
C2: Pruning 1stBT vs. Pruning 2ndBT	1	11.06^ns^	530^ns^	13.86^ns^	549.20^ns^
C3: Pruning vs. ‘Pruning+ PGR	1	120.72***	53^ns^	123.10***	54.41^ns^
Waste	115	6.85	154.52	8.69	198.35

^a^First branching tier

^b^: Second branching tier

^c^: Contrasts used for analysis of variance

^ns^Not significant

* Significance: *** = 0.001

** = 0.01; * = 0.05

The first contrast (C1) involved comparing the effects of pruning alone (regardless of the tier and application of growth regulators) and the control plots at the 1stBT and 2ndBT. The results revealed significant effects on the number of male and female flowers at the 1stBT. The second contrast (C2) compared the effects of pruning between the different tiers (1^st^ and 2^nd^), without considering the control. This contrast did not show a significant effect on increasing flower production. The third contrast (C3) compared the effects of ‘Pruning’ versus ‘Pruning + PGR’ showing a significant effect on NFF at both 1^st^ and 2^nd^ tiers (p<0.01).

**Fig 3:**
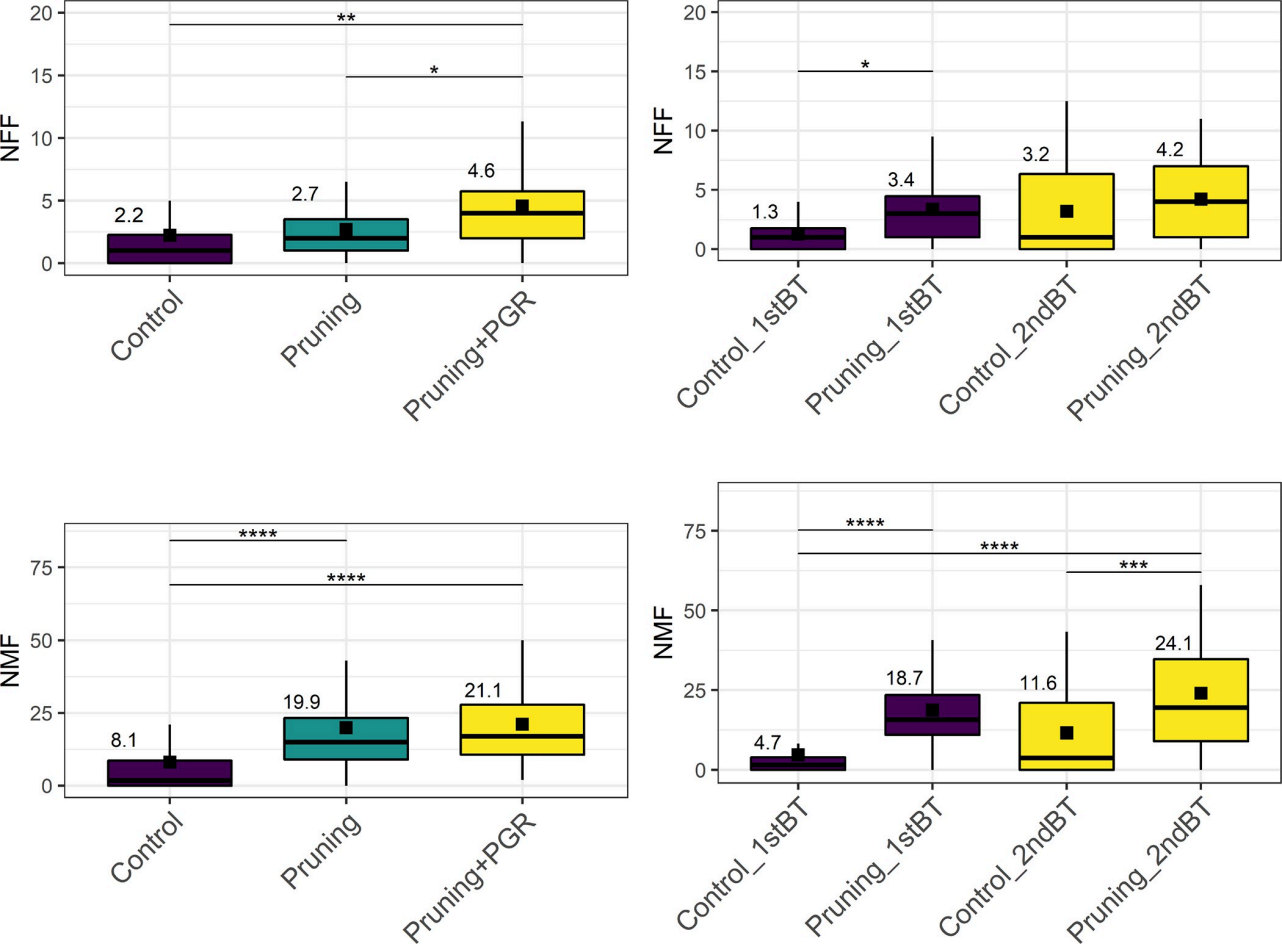
Boxplots of the means assessed for the characteristics: Number of female flowers (NFF) and number of male flowers (NMF) for the ’Control,’ ’Pruning,’ and ’Pruning + PGR’ treatments, evaluated at the first (1stBT) and second (2ndBT) branching levels. The upper lines represent statistically significant differences (p≤0.05) obtained by the Tukey test.

Fig 3 shows the means and distribution of BLUPs for the traits NFF and NMF for the treatments ‘Pruning’ and ‘Pruning + PGR’ in the two branch tiers evaluated. The use of pruning alone did not significantly increase flower production compared to the control for NFF. However, the combination of ‘Pruning + PGR’ increased the average number of flowers from 2.2 (control) to 4.6. Likewise, the use of ‘Pruning + PGR’ resulted in an increase in NMF from 8.1 to 21.1 when compared to the control. Although ‘Pruning’ and ‘Pruning + PGR’ produced similar results, the latter was able to provide an increase of approximately 10 flowers on average when compared to the control.

Both branching tiers evaluated showed significant differences for NFF and NMF when subjected to the ‘Pruning’ treatments for 1stBT (Fig 3). For NFF, there were statistical differences when pruning was performed at the 1stBT. As for NMF, there was a significant difference when pruning was performed at 1st or 2nd tier. The increase in NMF from pruning in 1stBT was 4.7 (control) to 18.7, and from pruning in 2ndBT it increased to 24.1.

**Fig 4:**
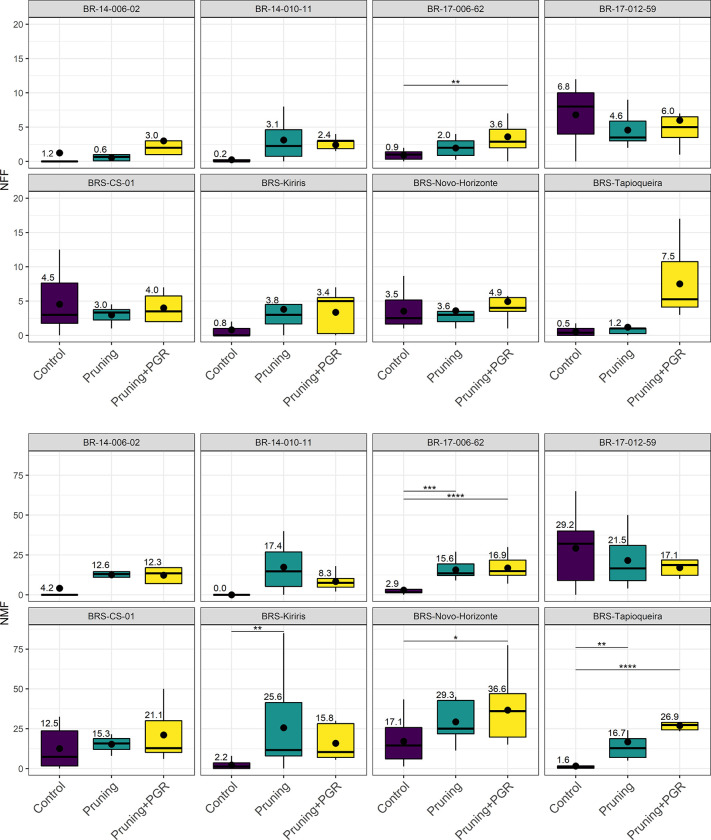
Boxplots of the individual performance of the eight cassava genotypes for the characteristics: Number of female flowers (NFF) and number of male flowers (NMF), concerning the pruning treatments, pruning with the use of plant growth regulators (PGR). The upper lines represent statistical differences by the Tukey test (p≤0.05).

Regarding the individual performance of the genotypes in the treatments involving the use of pruning, the study found that only the genotype BR-17-006-62 exhibited significant effects in increasing both NFF and NMF when subjected to the ‘Pruning + PGR’ treatment (Fig 4). The average increase in NFF was from 0.9 (control) to 3.6 (‘Pruning + PGR’), while the average number of flowers for NMF was from 2.9 (control) to 16.9 (‘Pruning + PGR’).

BRS Tapioqueira showed a superior response to the ‘Pruning + PGR’ treatment compared to the control and the ‘Pruning’ treatment, resulting in an average increase from 1.6 (control) to 26.9 male flowers (Fig 4). A similar trend was observed in BRS Novo Horizonte, with an increase from 17.1 (control) to 36.6 male flowers. On the other hand, the BRS Kiriris variety displayed positive effects in increasing the number of male flowers only with the use of pruning, with an average increase of approximately 23 flowers.

**Fig 5:**
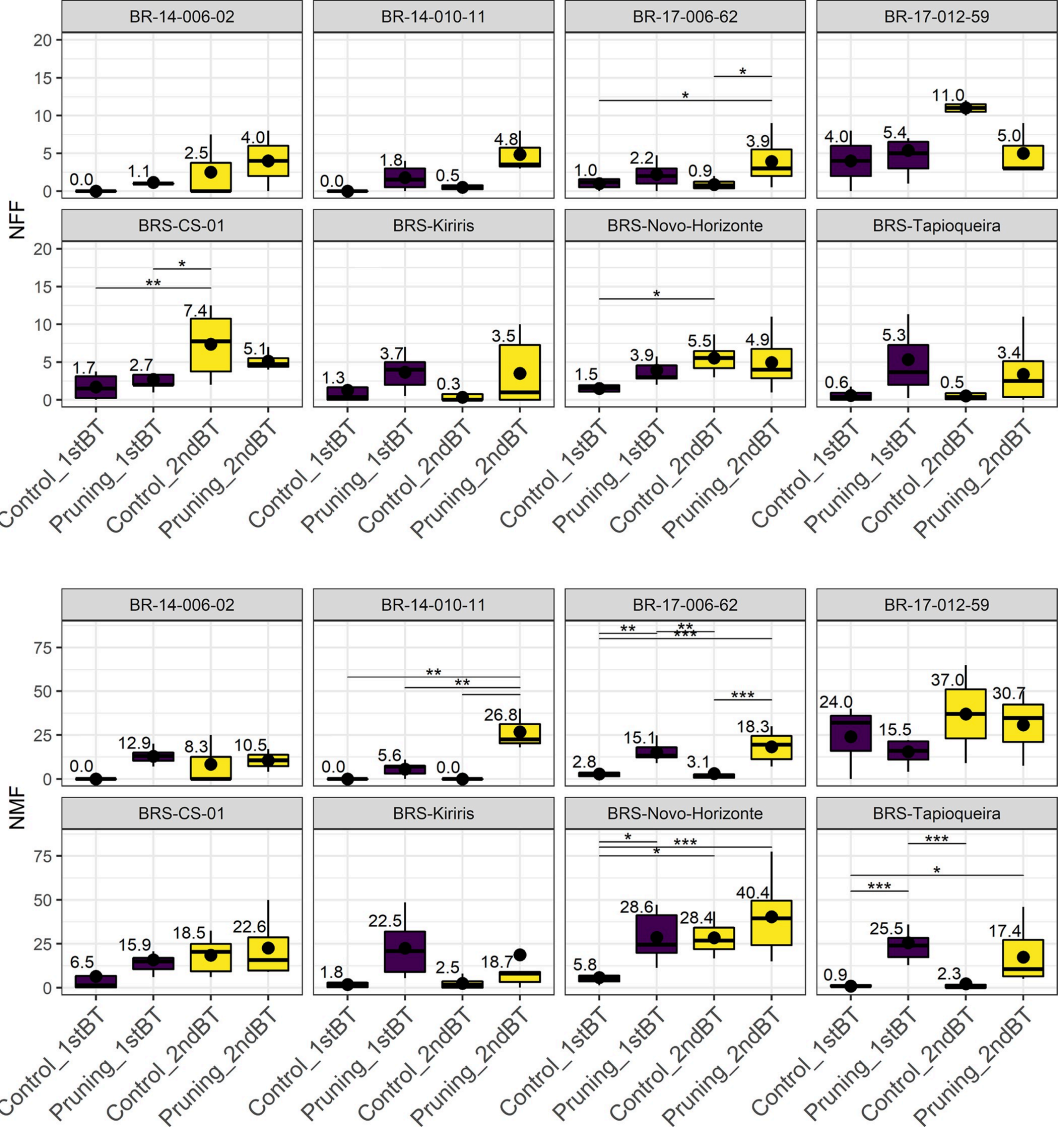
Boxplots of individual performance of pruning in the eight cassava genotypes for the characteristics: Number of female flowers (NFF) and number of male flowers (NMF). The colors differentiate the evaluated branching levels (1stBT and 2ndBT). The upper lines represent statistical differences by the Tukey test (p≤0.05).

The evaluation of the genotypes in terms of the ideal branching tier for inducing flowering showed that, in general, the 1stBT was sufficient to promote an increase in some characteristics associated with flowering, specifically NMF for the genotypes BR-17-006-62, BRS Novo Horizonte, and BRS Tapioqueira (Fig 5).

At the 2ndBT, one of the late and low flowering genotypes (BR-17-006-62) showed a significant increase in NFF with the use of pruning, resulting in an average increase from 2.8 (1stBT control) and 0.9 (2ndBT control) to 3.9 flowers (2ndBT pruning) (Fig 5). For the NMF trait, pruning at 2ndBT provided a 20% increase in the number of flowers for the BR-14-010-11 genotype, which did not produce any flowers in the controls, compared to pruning at 1stBT and 2nBT (26.8). Similar results were observed in BRS Tapioqueira and BR-17-006-62, which increased from 0.98 (1stBT control) and 2.3 (2ndBT control) to 17.4 flowers (2ndBT pruning).

## Discussion

### Action of photoperiod on flowering, branching levels, and flower production in cassava

The synchronization of flowering time and non-flowering of elite genotypes are still significant challenges in the recombination of cassava parents with favorable traits [47]. Among the genotypes evaluated, 50% showed a considerable reduction in the number of days to the onset of flowering, highlighting the positive influence of photoperiod extension on early flowering of cassava. In a related study on the phenology of cassava flowering, the highest percentage of flowering (59% of genotypes) was observed during the spring period, approximately six months after planting, which is characterized by longer photoperiods (12.34 hours) [12].

The inductive effect of photoperiod is closely related to the overexpression of genes and their role in regulating photoperiodism. Photoperiodism is the plant’s response to changes in day length and how it triggers flowering in response to circadian rhythms in the presence of light [22, 48]. The *t* locus (FT) gene is a crucial component of the flowering pathway in cassava, and its regulation is influenced by signaling components such as photoperiod and temperature [17]. Studies in *Arabidopsis* have demonstrated that photoperiod oscillation can either activate or repress FT gene regulators [49]. Additionally, in cassava, the presence of two homologs of FT, MeFT1, and MeFT2, play a crucial role in regulating flowering through photoperiod, with the expression of MeFT2 being directly dependent on the available photoperiod [17].

Flowering in cassava is closely linked with the number of branching tiers, as genotypes with more branching tend to have more flowers per plant [50]. In this study, extending the photoperiod significantly increased the number of branching tiers (NOB) for up to 240 DAP. The genotype BR-17-006-62, previously classified as having low-intensity late flowering, showed a higher likelihood of flowering (twice as much on average compared to the control) with the use of photoperiod extension. This agrees with [11] who reported that erect, low-flowering cassava genotypes increased branching levels from 0.58 to 2.60 under extended photoperiod.

While the extended photoperiod had positive effects on several flowering-related traits in cassava, it did not lead to an increase in the number of male and female flowers when only the first two branching tiers were considered. This finding contrasts with studies that reported higher flower production induced by photoperiod extension. In Colombia, where temperatures range from 30.1° ± 2.7°C throughout the year, a field experiment found that cassava plants exposed to 0.02 μmol of photons m^−2^ s^−1^ during the night with red light showed increased branching tiers and flower numbers [11]. [17] also reported that extending the photoperiod for 14 hours with white light and temperature ranging from 22° to 28°C enabled higher flowering levels in cassava genotypes. Moreover, photoperiod extension with red and blue LEDs in a 1:5 ratio induced flower induction and development in strawberry plants grown in a greenhouse for 14 hours at temperatures of 18° to 24°C in Canada [51].

While there are reports of successful photoperiod extension inducing flowering in cassava, some studies have also reported the inhibition of flowering when using photoperiod extension strategies. [52] reported a delay in floral initiation for the Ankpa4 genotype of *Vigna subterranea*, as well as a reduction in the number of flowers produced per plant, in response to different photoperiod extension treatments. Furthermore, [53] reported that photoperiod had a low influence on flowering in 37 woody species grown in subtropical areas with a rainfall of 1787 mm.

The low effect of photoperiod extension on male and female flower production observed in the present study may be attributed to the soil water control throughout the experiment, especially during dry periods when no supplemental irrigation was performed. Extended photoperiod increased flowering and fruiting in cassava when soil moisture was maintained through supplemental irrigation [11]. In our study, we aimed to evaluate the effects of treatments (photoperiod, pruning, and PGR) under natural growing conditions in the Coastal Tablelands region of Brazil. In this case, the largest flowering periods coincided with the highest rainfall observed throughout the year, from April to July, while during dry months (December to February), flower production was greatly reduced (S4 Fig).

In a comparative study conducted in two cities in Nigeria (Ubiaja and Ibadan), it was observed that the rate of flowering in cassava plants increased during periods of higher rainfall [25]. These findings suggest that the rainfall regime at the site is a contributing factor in explaining the differences in cassava flowering in various environments. It is hypothesized that the onset of rains, which results in a reduction in temperature, stimulates the onset of flowering in cassava in the tropics [54]. Therefore, in addition to using treatments to promote flowering in cassava, it is crucial to set up crossing blocks in regions or areas with irrigation potential during the drier periods of the year to avoid the suppression of the effects of other treatments.

### Effect of pruning and plant growth regulators on flower production in cassava

Pruning had a positive effect on flower production in the first two branching tiers of the plant. Early pruning is known to induce flowering by inhibiting the unrestrained growth of vegetative branches, which in turn can inhibit the growth of inflorescences and lead to their abortion. Furthermore, pruning has been found to increase the expression levels of homologs of IAA16 (a repressor of auxin responses) and TIFY10b (a transcription factor for jasmonic acid regulator) proteins, indicating an interaction between hormone signaling genes and pruning [25].

Pruning in the 2nBT resulted in a higher NFF and NFM. Furthermore, early pruning of the genotype BR17-006-62, which is characterized by low production of flowers, resulted in a 23% increase in the number of male flowers. Similar results have been reported in other studies, indicating that pruning can increase flower production in late-flowering genotypes with low seed production [11]. In pomegranate, vegetative growth immediately after pruning is associated with the activation of the PgCENa gene, which is a flowering suppressor of the phosphatidylethanolamine-binding protein (PEBP) family. On the other hand, a reduction in growth is associated with immediate flowering [55]. These findings suggest that the effect of pruning on flower production may vary depending on the species and genotype, and that the underlying molecular mechanisms may differ as well.

The results showed that pruning alone did not significantly increase the number of female flowers, but the combination of pruning with PGR resulted in a significant increase from 2.24 (control) to 5.19 female flowers. The flowering process is complex and involves various endogenous and exogenous factors that stimulate changes from vegetative to reproductive state in the apical meristem [22, 48]. Hormones such as GA, jasmonic acid, ethylene, ABA and their interactions with DELLA proteins play a significant role in the regulation of flowering [56]. The combination of pruning and the use of PGR such as benzyladenine (BA) and silver thiosulfate (STS) proved to be efficient in increasing the number of female flowers. Pruning and STS act to increase the number of flowers, while BA helps in promoting flower feminization. The effectiveness of PGR in delaying or accelerating flowering depends on the species, type, and concentration of the growth regulator used. In *Laelia anceps*, the use of BA and GA3 significantly reduced the number of days to the beginning of flowering and increased the number of stems and flowers [57]. In cassava, STS prevented inflorescence abortion and increase flower durability [16]. Additionally, the association between pruning and PGR facilitated female flower and fruit development, highlighting the role of hormonal factors and signaling pathways in floral development in cassava [25].

The number of flowers in cassava plants tends to increase with the number of branches. A study conducted by [50] revealed that male flowers were more abundant in all branches, while female flowers were more prevalent in the second and third branching tiers. Therefore, it is crucial to identify the optimal time to use invasive methods, such as pruning, to induce flowering and maximize the number of flowers within a given timeframe. Our findings recommend pruning the cassava plants from 2nBT, even if this means delaying the planned treatments and crossings for another 60 days. This approach is likely to result in a higher yield of flowers, thereby increasing the overall productivity of cassava plants.

### Prospects for cassava breeding

Although we did not observe a direct effect of photoperiod extension on cassava’s flowering and fruit set, our study found that extending the photoperiod increased flowering precocity, resulting in a 35-day reduction in the onset of flowering. Additionally, the use of PGR significantly increased the number of female flowers. Future studies investigating the molecular mechanisms responsible for the influence of BA on flower feminization and STS on the overall increase in flower number in cassava could help identify genes involved in this process. A recent study by [25] found that BA+STS treatment decreased the expression of cassava homologs such as ABI1 (Abscisic Acid Insensitive 1) and HG1 (Abscisic Acid Hypersensitive Germination 1). In contrast, pruning stimulated the expression of IAA16 and TIFY10b genes, which act as a repressor of auxin responses and regulator of jasmonic acid responses, respectively. Therefore, a positive correlation between these hormones signaling genes and pruning is suggested [58].

Combining treatments such as ‘Pruning + PGR’ with extended photoperiods can offer a viable alternative for reducing the initiation of flowering and increasing flower production in cassava. However, it will be necessary to validate our results in blocks of crosses with a greater number of elite genotypes from the breeding program, which generally have varied behavior regarding the initiation of flowering and number of flowers produced.

The synchronization of flowering in crossing blocks has been a significant challenge in improving cassava species, particularly when aiming to obtain progenies with wide recombination between specific chromosomal segments or breaking undesirable genetic links between certain traits. Therefore, increasing our understanding of the environmental and endogenous factors that regulate flowering time in cassava and their underlying molecular mechanisms is crucial. This understanding can help accelerate and enable the synchronization and maximization of flowering among cassava genotypes, thus improving the efficiency and effectiveness of cassava breeding programs.

## Final remarks

Our study demonstrates the potential of innovative approaches such as extending the photoperiod, premature pruning of branches, and using growth regulators to promote flowering induction in cassava. the extended photoperiod not only increases earlier flowering, but also enhances branch production. In contrast, pruning and growth regulators significantly increase the number of flowers produced. Moreover, growth regulators induce feminization, leading to an increase in the number of female flowers. These results offer valuable insights into optimizing the production of flowers required for crosses, allowing for an increase in the production of individuals per progeny.

## Supporting information

S1 Fig Rainfall, minimum, maximum and average monthly temperature, recorded from August 2020 to August 2021 in the experimental area of Embrapa Mandioca e Fruticultura (Cruz das Almas, BA, Brazil). (TIF)

S2 Fig Experimental drawing, the red circles represent the reflectors installed. The abbreviations mean the respective treatments, T1: no pruning; T2: pruning in the 1^st^ branching tier (1stBT); T3: pruning in the 2^nd^ branching tier (2nBT); T4: ‘Pruning + PGR’ (plant growth regulator) in the 1stBT; and T5: ‘Pruning + PGR’ in the 2ndBT.
(TIF)

S3 Fig Representation of the first and second branches in cassava. (TIF)

S4 Fig Averages of the number of female and male flowers, rainfall and minimum, maximum and average monthly temperature, recorded from August 2020 to August 2021 in the experimental area of Embrapa Mandioca and Fruticultura (Cruz das Almas-BA, Brazil). (TIF)
