# Supplementary figures and images for: Flowering induction in cassava using photoperiod extension premature pruning and plant growth regulators
Source: PLoS One. 2023 Oct 5;18(10):e0292385. doi: 10.1371/journal.pone.0292385 (PMC10553807; doi:10.1371/journal.pone.0292385)

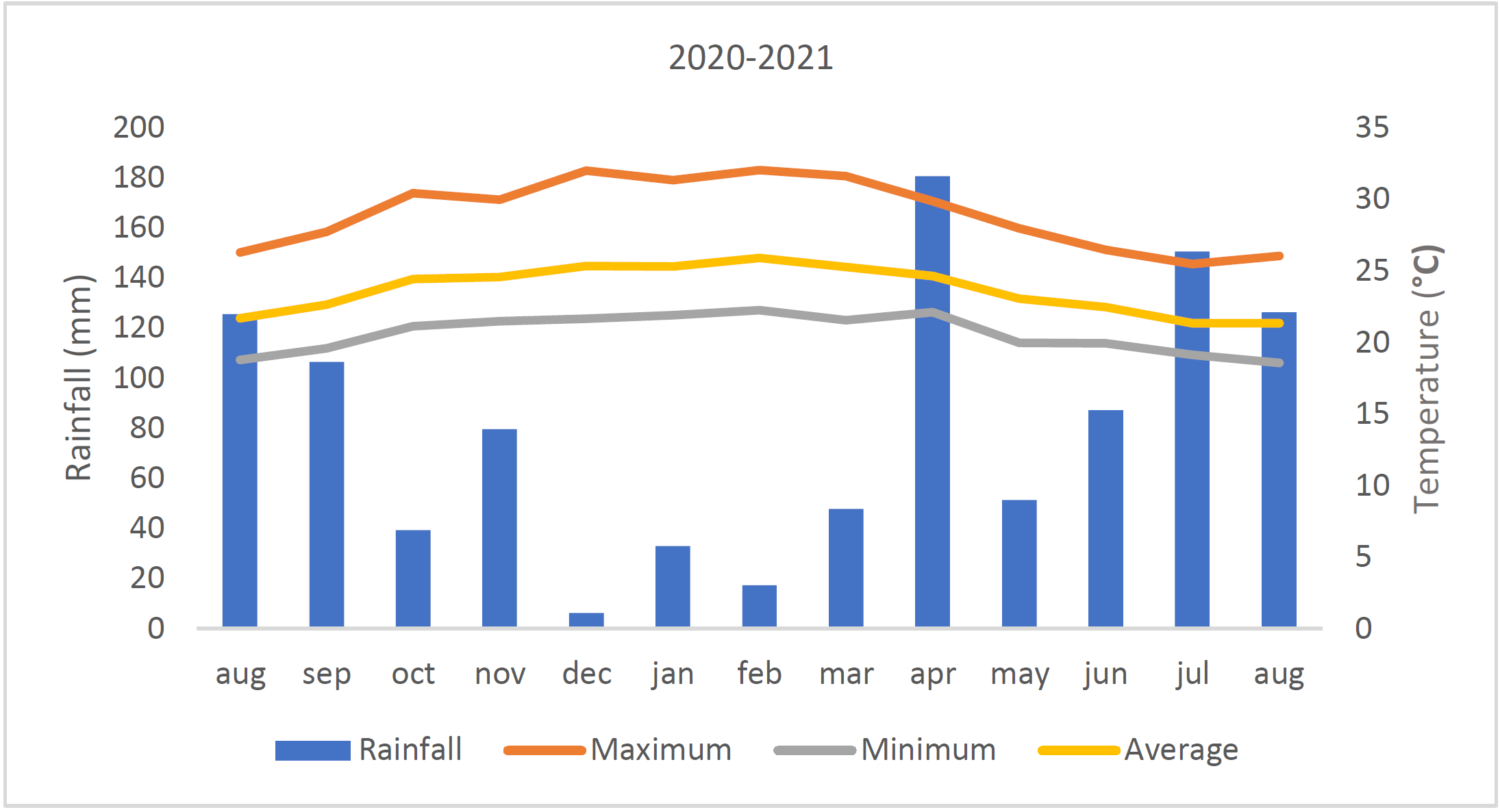

Supplement: S1 Fig — (TIF) [file pone.0292385.s001.tif]

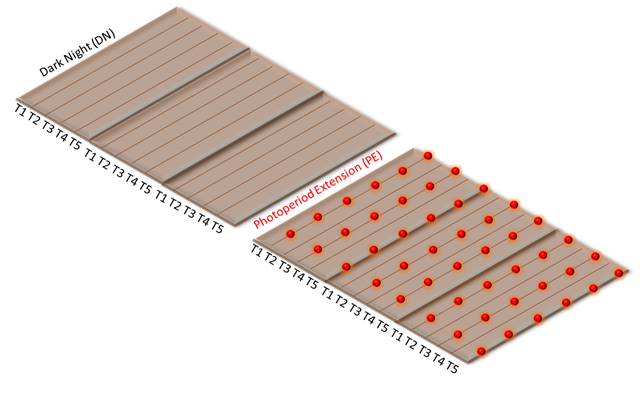

Supplement: S2 Fig — The abbreviations mean the respective treatments, T1: no pruning; T2: pruning in the 1st branching tier (1stBT); T3: pruning in the 2nd branching tier (2nBT); T4: ‘Pruning + PGR’ (plant growth regulator) in the 1stBT; and T5: ‘Pruning + PGR’ in the 2ndBT. (TIF) [file pone.0292385.s002.tif]

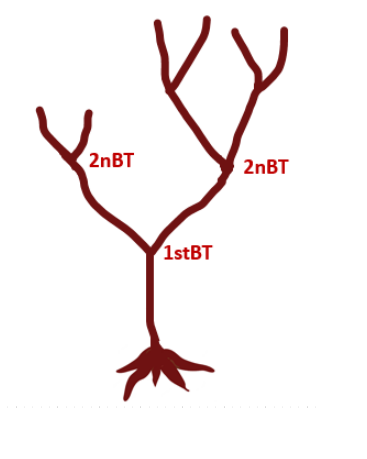

Supplement: S3 Fig — (TIF) [file pone.0292385.s003.tif]

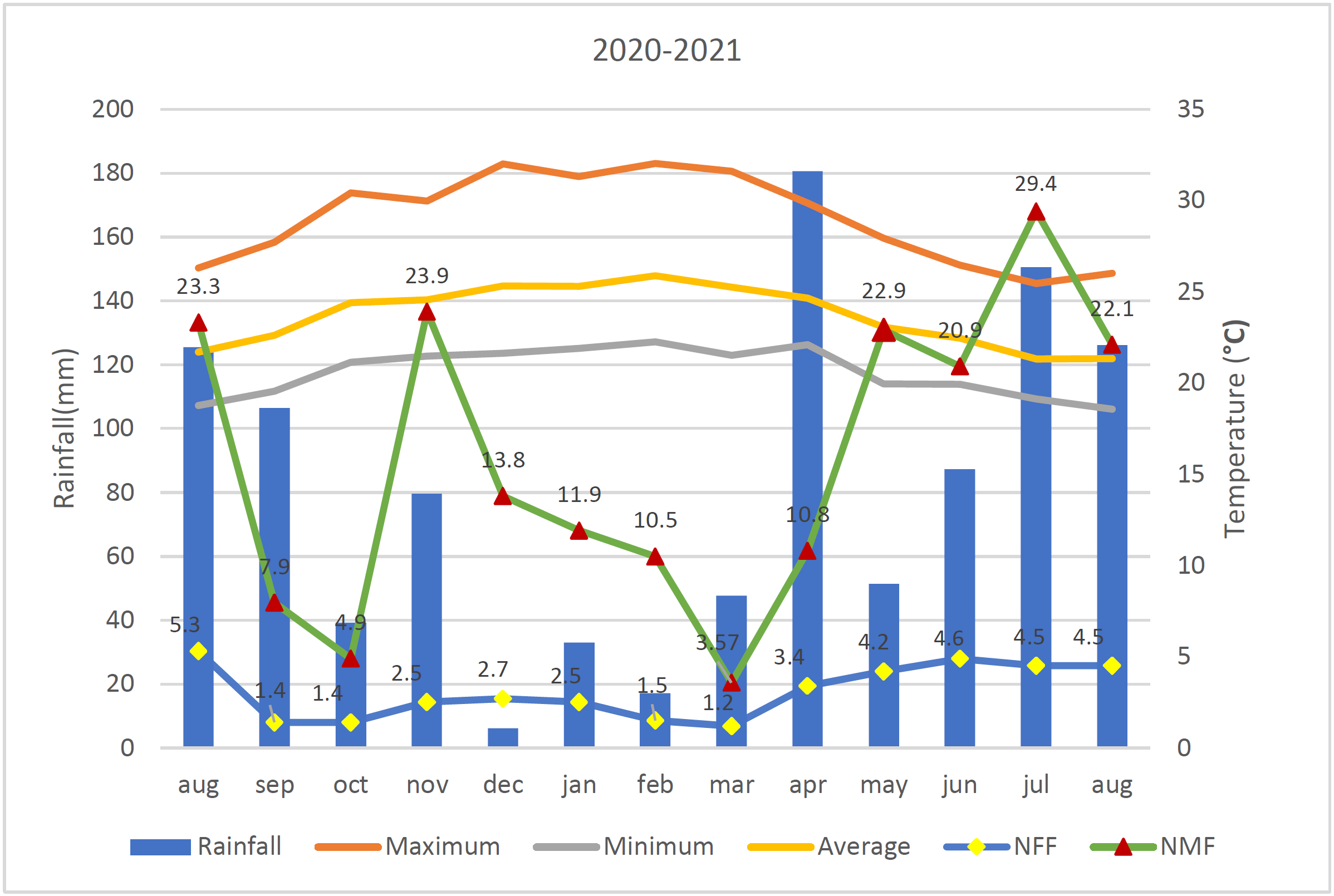

Supplement: S4 Fig — (TIF) [file pone.0292385.s004.tif]
